# Supplementary material for: Integrating physical and tactical factors in football using positional data: a systematic review
Source: PeerJ. 2022 Nov 14;10:e14381. doi: 10.7717/peerj.14381 (PMC9671036; doi:10.7717/peerj.14381)
Supplement: Supplemental Information 2 [file peerj-10-14381-s002.pdf]

International Platform of Registered Systematic Review and Meta-analysis Protocols

INPLASY PROTOCOL

To cite: Teixeira et al. Integrating physical, physiological and tactical factors in football using positional data: A systematic review. Inplasy protocol 202270030. doi: 10.37766/inplasy2022.7.0030

Received: 06 July 2022

Published: 06 July 2022

Corresponding author:
José Eduardo Teixeira

jose.eduardo@ipb.pt

Author Affiliation:
Instituto Politécnico de Bragança (IPB).

Support: **FCT - Project UIDB04045/2021.**

Review Stage at time of this submission: **Data analysis.**

Conflicts of interest:
None declared.

Integrating physical, physiological and tactical factors in football using positional data: A systematic review

Teixeira, JE[1]; Forte, P[2]; Ferraz, R[3]; Branquinho, L[4]; Silva, AJ[5]; Monteiro, AM[6]; Barbosa, TM[7].

Review question / Objective: **Positional data have been used to capture physical, physiological and tactical factors in football. However, previous systematic reviews captured the tactical behaviours without considering the integration of the different performance dimensions.**
Condition being studied: **Physical, physiological and tactical factors by positional data in in football.**
Information sources: **The Preferred Reporting Items for Systematic Reviews and Meta-Analyses (PRISMA) guidelines and the Population-Intervention-Comparators-Outcomes (PICOS) design were followed to conduct this systematic review (Malone et al., 2018; Moher et al., 2009). The literature search was based on three databases: PubMed/Medline, Web of Science (WoS, including all Web of Science Core Collection: Citation Indexes), and SportDiscus.**

INPLASY registration number: This protocol was registered with the International Platform of Registered Systematic Review and Meta-Analysis Protocols (INPLASY) on 06 July 2022 and was last updated on 06 July 2022 (registration number INPLASY202270030).

INTRODUCTION

Review question / Objective: **Positional data have been used to capture physical, physiological and tactical factors in football. However, previous systematic reviews captured the tactical behaviours without considering the integration of the different performance dimensions.**

Rationale: **To the best of our knowledge, no attempts have been made to review the state of art concerning that included integrative approaches to emphasize the**

Teixeira et al. Inplasy protocol 202270030. doi:10.37766/inplasy2022.7.0030 Downloaded from https://inplasy.com/inplasy-2022-7-0030/

positional data collected through tracking systems regarding physical, physiological and tactical variables. Also, a lack of the absence of procedural standardization to apply positional data in an integrative approach (Teixeira et al., 2022).

Condition being studied: Physical, physiological and tactical factors by positional data in in football.

METHODS

Search strategy: According to the search strategy were included studies from January 2000 for relevant publications using keywords in table 1. In addition, the study variables are a Boolean search phrase (Table 1). The literature search was accessed during April and May 2022. The search strategy was independently conducted by one review author and checked by a second author. Discrepancies between the authors in the study selection were solved with support a third reviewer. The authors did not prioritize authors or journals. The search was limited to peer-reviewed original articles published online until May 2022. Duplicate articles were identified and eliminated prior to application of the selection criteria. Titles and abstracts were initially selected and excluded according selection criteria. The selection of full texts articles was based on a selection to determine the final status: inclusion and exclusion criteria. Disagreements were resolved through discussion between two authors, or via a third researcher if required. Secondary-sourced articles considered relevant and with same screening procedures have been added.

Participant or population: Adult and youth football players.

Intervention: Integration of physical/ physiological and tactical measures using spatiotemporal datasets.

Comparator: Physical, physiological and tactical variables.

Study designs to be included: Observational cohort and randomized controlled trial (RCT).

Eligibility criteria: The studies included in the present review followed the following inclusion criteria: (1) original article focused on adult and youth football players of both sexes; (2) studies with screening procedures based on physical, physiological and tactical measures using tracking and positional data; (3) studies that used spatiotemporal to assess physical and physiological; (4) studies that used positional and tracking data to measures spatiotemporal and tactical variables through time-series; (3) other performance factors as psychophysiological, technical and contextual factors were not excluded from the present review only if both variables of interest (i.e. physical, physiological and tactical measures) were also part of the experimental design; (5) studies of human physical and physiological performance in Sport Science and as scope; (6) original article published in a peer-review journal; (7) full text available in English; (8) article reported sample and screening procedures (e.g. data collection, study design, instruments, and the outcomes).The exclusion criteria were: (1) original articles individual and others team sports as football code population (e.g. Australian Football, Gaelic Football, Union and/or Seven Rugby); (2) studies that study that analysed none or only one of the target performance factors (i.e. physical/ physiological or tactical measures); (3) studies which integrate several performance factors, but do not combine the two variables of interest even if one of them is integrated; (4) studies that measured physical and physiological using on field based test and laboratory test rather than tracking and positional data; (5) studies that included tactical variables using notational analysis and other methodological procedures that do not assess spatiotemporal and positional data through time-series; (6) others research areas and non-human participants; (7) articles with bad quality in the description of study sample and screening procedures

Teixeira et al. Inplasy protocol 202270030. doi:10.37766/inplasy2022.7.0030 Downloaded from https://inplasy.com/inplasy-2022-7-0030/

(e.g. data collection, study design, instruments, and the measures) according to PEDro and Downs and Black scales; (8) reviews, abstract/papers conference, surveys, opinion pieces, commentaries, books, periodicals, editorials, case studies, non-peer-reviewed text, masters and/or doctoral thesis.

Information sources: The Preferred Reporting Items for Systematic Reviews and Meta-Analyses (PRISMA) guidelines and the Population-Intervention-Comparators-Outcomes (PICOS) design were followed to conduct this systematic review (Malone et al., 2018; Moher et al., 2009). The literature search was based on three databases: PubMed/Medline, Web of Science (WoS, including all Web of Science Core Collection: Citation Indexes), and SportDiscus.

Main outcome(s): Not described.

Additional outcome(s): Not described.

Data management: The data extractions from the included articles were performed according to Teixeira et al. (2021): (1) summary measures describing performance dimension, measure, measurement, thresholds and/or metric formula with included article reference and further reading; (2) sampling characteristics according to publication date, study design, population, competitive level, sample (N), sex, age, expertise level and quality assessment; (3) characterisation of the research methodology was performed by study purpose, game format experimental approach, methodological procedures, data collection, statistical and mathematical analysis. The outcome measures and the statistical procedures used in the included references were inconsistent between studies, making it impossible to grouped data and perform the meta-analysis. Characterization of participants is reported as mean ± standard deviation, confidence interval (IC) and effect size (ES) wherever possible.

Quality assessment / Risk of bias analysis: The methodological quality was assessed using the modified Downs and Black Quality Index (cross-sectional studies) and the Physiotherapy Evidence Database (PEDro) scale (intervention studies).

Strategy of data synthesis: The data extractions from the included articles were performed according to Teixeira et al. (2021): (1) summary measures describing performance dimension, measure, measurement, thresholds and/or metric formula with included article reference and further reading; (2) sampling characteristics according to publication date, study design, population, competitive level, sample (N), sex, age, expertise level and quality assessment; (3) characterization of the research methodology was performed by study purpose, game format experimental approach, methodological procedures, data collection, statistical and mathematical analysis.

Subgroup analysis: Physical/Physiological data, positional data and other dimensions (i.e., technical, tactical and psychophysiological factors).

Sensitivity analysis: Not described.

Language: English.

Country(ies) involved: Portugal.

Keywords: tracking systems, movement, complexity, training, match.

Contributions of each author:
Author 1 - José E. Teixeira - Conceptualization, data curation, investigation, software, and writing – original draft.
Email: jose.eduardo@ipb.pt
Author 2 - Pedro Forte - Conceptualization, data curation, formal analysis, methodology, resources, and writing – review & editing.
Email: pedromiguel.forte@iscedouro.pt

Teixeira et al. Inplasy protocol 202270030. doi:10.37766/inplasy2022.7.0030 Downloaded from https://inplasy.com/inplasy-2022-7-0030/

Author 3 - Ricardo Ferraz - Data curation, formal analysis, software, and writing – review & editing.
Email: rmpf@ubi.pt
Author 4 - Luís Branquinho - Formal analysis, investigation, methodology, software, and writing – review & editing.
Email: luis.branquinho@iscedouro.pt
Author 5 - António J. Silva - Supervision and writing – review & editing.
Email: ajsilva@utad.pt
Author 6 - António M. Monteiro - Resources, supervision, and writing – review & editing.
Email: mmonteiro@ipb.pt
Author 7 - Tiago M. Barbosa - Funding acquisition, methodology, resources, validation, and writing – review & editing.
Email: barbosa@ipb.pt

Teixeira et al. Inplasy protocol 202270030. doi:10.37766/inplasy2022.7.0030 Downloaded from https://inplasy.com/inplasy-2022-7-0030/
