# Supplementary material for: Integrating physical and tactical factors in football using positional data: a systematic review
Source: PeerJ. 2022 Nov 14;10:e14381. doi: 10.7717/peerj.14381 (PMC9671036; doi:10.7717/peerj.14381)
Supplement: Supplemental Information 3 [file peerj-10-14381-s003.docx]

**The rationale for conducting the systematic review / meta-analysis:**

Currently, a growing reviews and meta-analysis have been published on this research topic focusing on training and match insights (Low et al., 2020; Rago et al., 2020; Teixeira et al., 2021). However, reviewing articles have considered each performance factor independently, describing tactical behaviour separately from physical and physiological demands. Also, previous systematic reviews captured the tactical behaviours without considering the integration of the different performance dimensions (Coito et al., 2022; Ometto et al., 2018).

**The contribution that it makes to knowledge in light of previously published related reports, including other meta-analyses and systematic reviews:**

To the best of our knowledge, no attempts have been made to review the state of art concerning that included integrative approaches to emphasize the positional data collected through tracking systems regarding physical, physiological and tactical variables. Thus, a lack of the absence of procedural standardization to apply positional data in an integrative approach (Teixeira et al., 2022). Thus, the aim of this study was to systematically review the published articles that integrate physical, physiological and tactical variables in football using positional data.

Among the current systematic review, the physical and physiological data can be integrated by player’s movement speed, heart-rate based measures and perceived exertion. Positional datasets can be computed by spatial movement variability/regularity, complex index, coordination/synchronization using intra-team and inter-team dyads, playing space. Futures researches should consider applying positional data in women's football environments and explore the representativeness of the MSG and LSG.
